# Psychosocial Impact of COVID-19 Pandemic in Elderly Psychiatric Patients: a Longitudinal Study

**DOI:** 10.1007/s11126-021-09917-8

**Published:** 2021-04-26

**Authors:** Magdalena Seethaler, Sandra Just, Philip Stötzner, Felix Bermpohl, Eva Janina Brandl

**Affiliations:** Department of Psychiatry and Psychotherapy, Campus Charité Mitte (Psychiatric University Clinic at St. Hedwig Hospital), Charité-Universitätsmedizin Berlin, Corporate Member of Freie Universität Berlin, Humboldt-Universität zu Berlin, and Berlin Institute of Health, Berlin, Germany

**Keywords:** COVID-19, Elderly, Geriatric psychiatry, Mental health, Psychosocial burden, Depression

## Abstract

The study was designed to investigate the impact of the Coronavirus Disease 2019 (COVID-19) pandemic on mental health and perceived psychosocial support for elderly psychiatric patients in a longitudinal design. *n* = 32 patients with affective or anxiety disorders aged ≥60 years were included. Telephone interviews were conducted in April/May 2020 (T1) and August 2020 (T2). The psychosocial impact (PSI) of the pandemic and psychopathology were measured. Changes between T1 and T2 were examined. Patients’ psychosocial support system six months before the pandemic and at T1/T2 was assessed. We found a significant positive correlation between general PSI and depression as well as severity of illness. General PSI differed significantly depending on social contact. Neither general PSI nor psychopathology changed significantly between T1 and T2. At T1, patients’ psychosocial support systems were reduced as compared to six months before. Patients reported an increase in psychosocial support between T1 and T2 and high demand for additional support (sports, arts/occupational therapy, physiotherapy, psychotherapy). Elderly psychiatric patients show a negative PSI of the pandemic. They are likely to suffer from an impaired psychosocial situation, emphasizing the importance of developing concepts for sufficient psychosocial support during a pandemic.

## Introduction

Since the beginning of the Coronavirus Disease 2019 (COVID-19) pandemic in December 2019 in Wuhan, China, the Severe Acute Respiratory Syndrome Coronavirus 2 (SARS-CoV-2) has spread worldwide [1]. The overall cumulative hospitalization rate is described as 193.7 per 100,000, increasing to between 329.4 and 884.8 per 100,000 in people 65 years [2], who are considered a high-risk population [3].

Globally, more than one third of all COVID-19 patients were admitted to intensive care units (ICU) [4]. Older age was shown to be a risk factor for lethal course of COVID-19 [5]. Mortality increases to 14.8% to 28.7% in patients aged 80 years or older compared to an overall rate of 2.29% to 5.4% [3, 6].

Within a short period, SARS-CoV-2 has had an enormous impact on the world’s societies, health systems and economy. Policies such as movement restrictions, social distancing and isolation and the hazard and consequences of getting infected with SARS-CoV-2 are likely to impact mental health, as this has been found for past epidemics/pandemics, such as the Spanish flu, Ebola or the Middle East Respiratory Syndrome-related Coronavirus (MERS-CoV) outbreak in 2015 [7–12].

The current COVID-19 pandemic may entail similar or even more serious consequences [13, 14]. SARS-CoV-2 has caused more than 2.5 million deaths as of March 2021 and is still spreading and accelerating [1] – heading towards far-reaching repercussions.

Negative psychosocial effects of the COVID-19 pandemic and social distancing measures, including general distress, traumatization, anxiety and depression were reported [15–17].

Several studies have examined mental health of vulnerable populations during the pandemic, such as health workers or SARS-CoV-2 patients [18, 19]. Among health workers in China and Singapore, elevated symptoms of anxiety [20–22], stress [20–22], Posttraumatic Stress Disorder (PTSD) [22, 23], depression [21, 22] were reported. One previous study found a negative psychological impact of the COVID-19 pandemic on the general elderly population in the US [24] .

To our knowledge, there are no studies specifically examining the psychosocial impact (PSI) of the pandemic in elderly patients with pre-existing mental disorders. Especially the elderly are vulnerable and prone to showing severe illness from SARS-CoV-2 and its sequelae due to age and health conditions [25] and therefore being advised to follow precautionary measures such as highly restrictive social distancing [26]. Furthermore, for elderly people with an affective or anxiety disorder, fear of infection combined with reduction of social contacts (private and professional) might cause exacerbation of symptoms.

In this study, we systematically evaluated the impact of the COVID-19 pandemic on mental health of elderly patients with affective or anxiety disorders, investigating 1) *sociodemographic and clinical information,* 2) *psychosocial impact of the COVID-19 pandemic,* 3) *psychopathology* and 4) *psychosocial support*.

## Methods

### Participants

Sixty-eight patients were invited with a letter containing the study information and consent forms. In telephone calls, further information regarding the study was provided by the first authors. Thirty-three individuals provided written consent to participate. Reasons to refrain from participation included unstable mental health, hospitalization, and lack of interest in participation. *n* = 32 participants were finally included at T1 and *n* = 24 at T2 (see Table 1 for detailed sample characteristics). All participants were current or former patients of the Psychiatric University Hospital of Charité at St. Hedwig Hospital, Berlin, Germany. Participants were: (1) inpatients or outpatients with a main diagnosis of affective or anxiety disorder according to DSM-5 (Diagnostic and Statistical Manual of Mental Disorders) confirmed by trained clinicians; (2) showed native proficiency in German language; (3) showed no severe cognitive impairment; (4) were ≥ 60 years old. The study was approved by the local ethics committee and followed the principles of the Declaration of Helsinki. The work was conducted taking into consideration principles for research involving humans, such as the aim to increase knowledge, a given necessity, the expectation of benefits and a voluntarily consent by patients after providing suitable information [27].

**Table 1:** Sociodemographic data

Variables		*n* (%) T1/T2
Number of patients		32/24
Age		
	Minimum	60
	Maximum	93
	Mean ± Standard deviation	77,94 ± 8,12/78.25 ± 8.43
Sex		
	Female	20 (62.5%)/16 (66.67%)
	Male	12 (37.5%)/8 (33.33%)
Income		
	Pension	27 (84.38%)/20 (83.33%)
	Sick leave	1 (3.13%)/0
	Looking for work	2 (6.25%)/2 (8.33%)
	Other	2 (6.25%)/2 (8.33%)
Living situation		
	Rent	20 (62.5%)/18 (75%)
	Own property	4 (12.5%)/1 (4.2%)
	Nursing home	4 (12.5%)/2 (8.3%)
	Assisted living	1 (3.13%)/1 (4.2%)
	Other	2 (6.25%)/2 (8.3%)
	No information	1 (3.13%)/0 (0%)
Relationship status		
	None	8 (25%)/6 (25%)
	Married	9 (28.13%)/7 (29.2%)
	Widowed	8 (25%)/7 (29.2%)
	Divorced/separated	7 (21.88%)/4 (16.7%)
Household		
	Alone	17 (53.13%)/12 (50%)
	With partner	9 (28.13%)/6 (25%)
	With other relatives	1 (3.13%)/2 (8.3%)
	With multiple different relatives	5 (15.63%)/3 (12.5%)
	Assisted living	0 (0%)/1 (4.2%)
Social contact		
	Close	9 (28.13%)/7 (29.2%)
	Regularly	13 (40.63%)/10 (41.7%)
	Rarely	10 (31.25%)/7 (29.2%)

### Study Design

A longitudinal design with two measurement points was chosen since our study strives for a better understanding of potential long-term pandemic-related impairment of psychiatric conditions as well as patients’ psychosocial support system.

At the first measurement point T1 in April/May 2020, in Germany, schools and other institutions were closed and visits to hospitals/nursing homes were limited [28]. Further, many outpatient departments offered none or only digital appointments instead of face-to-face contacts. Especially for the elderly, this may have been challenging due to limited knowledge of and access to digital media [29]. Also, elderly patients often suffer from loneliness [30] and are more dependent on efficient care. Therefore, we expected to find a substantial negative PSI of the pandemic among elderly psychiatric patients at T1. The second measurement point T2 was scheduled three months after T1, in August 2020, to assess potential changes in PSI and psychopathology as well as patients’ private and professional support system.

We hypothesized that PSI may be reduced at T2 due to decreased infection numbers and restrictions and habituation to the pandemic situation after a “first wave”. Alternatively, considering the duration of 4 to 5 months of the pandemic with presumably ongoing containment measures or even facing a further escalated pandemic situation in Germany in August 2020, we assumed to find an increased PSI and overall deterioration of the psychosocial situation of elderly psychiatric patients.

### Procedure

Interviews with a duration of approximately 20 min were conducted via telephone by trained clinicians. T1 baseline interviews took place in April and May 2020. T2 was set in August 2020, over a period of 27 days.

### Measures

The structured interview consisted of five topics: 1) *sociodemographic and medical information*, including rating of social contact behavior on a scale from 0 (no contact) to 4 (close contact). 2.a) *psychosocial impact of the COVID-19 pandemic* was assessed using a questionnaire with 15 items (see Table 3) developed for this study, where participants rated the impact of the pandemic on several aspects from 0 (does not apply) to 5 (extremely high negative impact). 2.b) Additional open questions asking about what bothered participants most about the pandemic. 3) For *psychopathology*, depressive symptoms in the last two weeks (yes/no format) were assessed with the 15-item version of the German Geriatric Depression Scale (GDS-15) [31]. The GDS-15 has shown good psychometric criteria [32, 33] and its German version has been used in clinical research [34]. Scores range from 0 (no depression) to 15 (severe depression), a cut-off ≥5 is recommended [35]. Additional questions about general psychopathology included assessment of suicidality, anxiety, obsessive-compulsive disorder (OCD) and psychotic symptoms as well as substance abuse/addiction utilizing open questions according to the AMDP system [36]. Lifetime/current suicidality was rated as 1 (none), 2 (suicidal thoughts), 3 (suicidal plans), and 4 (suicidal attempt). Other psychopathology was assessed as 0 (none) or present (1). Severity of illness was assessed with the Clinical Global Impression – Severity Scale (CGI) [37] from 1 (not at all ill) to 7 (extremely severely ill). 4) Questions concerning *psychosocial support* asked whether participants received certain psychosocial support (see Table 4) in the six months prior to the pandemic (at T1) or since T1 (at T2) or were currently receiving them. If not, it was asked whether this was due to the pandemic and whether they wished to receive this form of support.

### Statistical Analysis

Statistical analyses were performed using IBM SPSS Statistics for Windows, Version 26.0 (Armonk, NY: IBM Corp.). Descriptive statistics and percentages were determined for all outcome variables. For group comparisons, assumptions for parametric tests were examined (normality as assessed by the Shapiro-Wilk-test and visual examination regarding outliers and distribution). Group differences were assessed using Kruskal-Wallis-tests, while analyses of differences between two groups were *t*-tests, Mann-Whitney *U* tests or *χ*^2^-tests. Dunn-Bonferroni multiple comparison correction was conducted. Paired tests were computed to compare scores in main outcome variables between T1 and T2, either using paired *t-*tests, Wilcoxon-signed-rank-test or McNemar’s test. Pearson product-moment correlation coefficients were computed to assess the relationship between psychological impact of the pandemic and other outcome variables. *P*-values below *p* < 0.05 were considered significant.

## Results

### Sample Characteristics

Sociodemographic characteristics of the sample both at T1 and T2 are presented in Table 1**.** There were no significant differences in characteristics between dropouts and non-dropouts – however, several patients explained dropping out with a high psychosocial burden of the COVID-19 pandemic itself.

None of the participants reported a SARS-CoV-2 infection or quarantine stay from six months before the start of the study until T2.

A significant life event since T1 was reported by *n* = 10 patients at T2. *n* = 3 patients underwent major surgery and *n* = 2 reported severe suicidal thoughts, one of them tried to commit suicide and was admitted to an inpatient facility. *n* = 5 patients reported other challenges.

Clinical characteristics and psychopathology are portrayed in Table 2.

**Table 2:** Psychopathology and Clinical Data

Variables		*n* (%) T1/T2
Main diagnosis		
	Depressive Episode (F32)	5 (15.63%)
	Recurrent Depressive Disorder (F33)	20 (62.5%)
	Anxiety Disorder (F4)	3 (9.38%)
	Bipolar Affective Disorder (F31)	3 (9.38%)
	Persistent Affective Disorder (F34)	1 (3.13%)
Secondary diagnosis		13 (40.63%)
Somatic diagnosis		
	With somatic diagnosis	28 (87.5%)
	Without somatic diagnosis	2 (6.26%)
	No information	2 (6.26%)
Medication		
	Antidepressant	13 (40.63%)
	Antipsychotic	2 (6.25%)
	Antidepressant and antipsychotic	4 (12.5%)
	Antidepressant and other	4 (12.5%)
	Antidepressant, antipsychotic and other	4 (12.5%)
	Other	3 (9.38%)
	Change in last 2 weeks	9 (28.13%)
	No change in last 2 weeks	21 (65.63%)
	Without medication	2 (6.26%)
CGI		
	Minimum	1
	Maximum	6
	Mean ± Standard deviation	3.16 ± 1.59/3.00 ± 1.41
GDS sum		
	Minimum	1
	Maximum	12/11
	Mean ± Standard deviation	5.66 ± 3.48/5.50 ± 3.62
Anxiety	Yes	19 (59.38%)/14 (58.33%)
	No	13 (40.63%)/10 (41.67%)
Lifetime suicidality	Never	11 (34.38%)
	Thoughts	11 (34.38%)
	Plans	4 (12.5%)
	Attempts	6 (18.75%)
Suicidality	No	21 (65.63%)/18 (75%)
	Thoughts	11 (34.38%)/4 (16.67%)
	Plans	0 (0)/ 1 (4.17)
	Attempts	0 (0) /1 (4.17)

### Psychosocial Impact

**Fig. 1:**
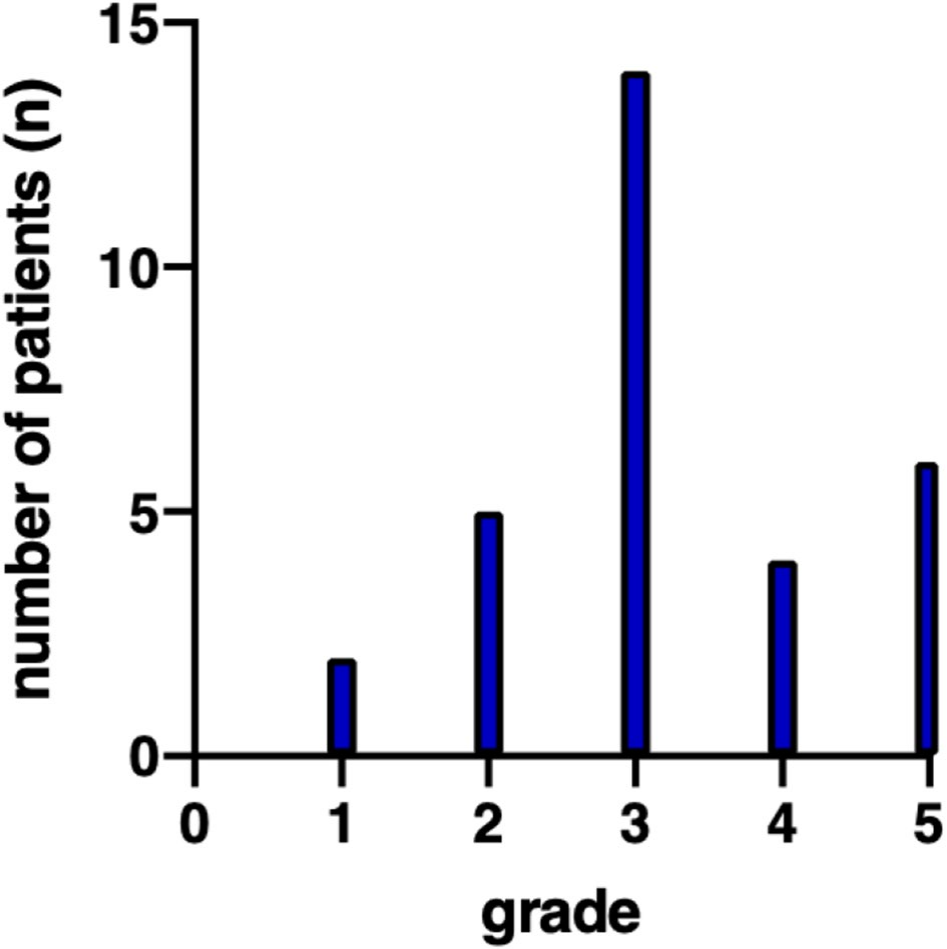
General PSI all patients. The general psychosocial impact (PSI) from 0 (does not apply) to 5 (extremely high negative impact) portrays the overall psychosocial burden due to the pandemic situation. The number of patients with certain general PSI values is shown

Mean PSI values were calculated for each question of the questionnaire, presented graphically for general PSI in Fig. 1 (item 1: “How negatively impacted have you felt in the last two weeks generally due to the COVID-19 pandemic?”).

Three patient groups were formed depending on patients’ social contact behavior at T1: *n* = 9 patients (28.13%) reported close social contact, *n* = 13 patients (40.63%) regular and *n* = 10 patients (31.25%) rare social contact.

At T1, a significant difference in general PSI values between patients with close (Mdn = 4.00), regular (Mdn = 3.00) and rare social contact (Mdn = 3.00; H(2) = 6.366; *p* = .041) was found. Dunn-Bonferroni post-hoc analysis revealed a significantly higher general PSI in patients with close as compared to regular social contact (*p* = .013). At T2, no significant difference in general PSI values between patients with close (Mdn = 3.00), regular (Mdn = 3.00) and rare social contact (Mdn = 3.00; H(2) = 1.038; *p* = .595) was shown. PSI values were lowest in patients with regular social contact at T1 as well as T2.

At T1, there was a significant positive correlation between general PSI and GDS-15 sum scores (*r*(30) = .384, *p* = .030) and between general PSI and CGI values (*r*(30) = .376, *p* = .034). While these correlation coefficients were non-significant at T2, several other significant correlations were found between psychosocial impact and other outcome variables. Table 3 gives an overview of correlation coefficients at T1 and T2 between the 15 PSI items and the outcome variables: GDS-15 sum score, CGI value, current suicidality and social contact behavior.

**Table 3:** Pearson correlation coefficients between PSI and main outcome variables

How negatively impacted have you felt in the last two weeks…		GDS-15 sum score		CGI		current suicidality		social contact behavior
		T1	T2	T1	T2	T1	T2	T1
…generally due to the COVID-19 pandemic?	T1	.384*	–	.376^*^	–	.086	–	−.330
	T2	–	.286	–	.141	–	.226	–
…because you belong to the risk group due to pre-existing somatic conditions?	T1	.456^**^	–	.202	–	−.098	–	−.357^*^
	T2	–	.650**	–	.377	–	.346	–
…because you had symptoms possibly related to COVID-19?	T1	.210	–	.199	–	.122	–	.081
	T2	–	n/a	–	n/a	–	n/a	–
…because people close to you had symptoms possibly related to COVID?	T1	−.053	–	.079	–	.196	–	−.201
	T2	–	n/a	–	n/a	–	n/a	–
…due to difficulties in receiving planned medical care/medication?	T1	.256	–	.158	–	−.205	–	−.401^*^
	T2	–	.200	–	.252	–	.173	–
…due to difficulties in receiving unplanned medical care/medication?	T1	.225	–	.149	–	−.011	–	−.327
	T2	–	.110	–	.174	–	.031	–
…due to lockdown restrictions?	T1	.577^**^	–	.638^**^	–	.155	–	−.231
	T2	–	.341	–	.363	–	.371	–
…due to social/family conflicts?	T1	.400^*^	–	.345	–	.184	–	.197
	T2	–	.049	–	.125	–	−.019	–
…due to restrictions concerning leisure and social activities?	T1	.286	–	.327	–	−.048	–	−.368^*^
	T2	–	.550*	–	.461*	–	.343	–
…because you had financial problems due to the COVID-19 pandemic?	T1	.204	–	.378^*^	–	.014	–	−.293
	T2	–	.151	–	.238	–	.260	–
…due to difficulties in getting daily necessities?	T1	.217	–	.270	–	.139	–	−.082
	T2	–	.039	–	.168	–	.185	–
…due to difficulties in shopping not related to daily necessities?	T1	.293	–	.089	–	−.272	–	−.408^*^
	T2	–	.447*	–	.330	–	−.062	–
…because people close to you were negatively impacted by the COVID-19 pandemic?	T1	.391^*^	–	.439^*^	–	.077	–	−.260
	T2	–	.355	–	.337	–	.512*	–
…due to the media coverage of the COVID-19 pandemic?	T1	.146	–	−.016	–	−.047	–	−.056
	T2	–	.265	–	.066	–	.161	–
…because you did not receive enough information about the COVID-19 pandemic?	T1	.387^*^	–	.203	–	.071	–	.253
	T2	–	.275	–	.183	–	.540**	–

The values in boldface actually represent correlation coefficients and not p-values. They appear in boldface if the p-value behind the result is * p < .05 or ** p < .001 respectively;

T1: *n* = 32, *df* = 30; T2: *n* = 24, *df* = 22;

n/a = could not be computed because PSI variable was constant

*PSI* Psychosocial Impact, *GDS-15* German Geriatric Depression Scale, *CGI* Clinical Global Impression

### Changes between T1 and T2

Comparing results of the PSI questionnaire at T1 and T2 revealed that the negative PSI due to lockdown restrictions was significantly higher at T1 (*M* = 2.46; *SD* = 1.82) as compared to T2 (*M* = 1.42; *SD* = 1.86), *t*(23) = 2.389, *p* = .025. The negative PSI due to difficulties in shopping for goods other than daily necessities was significantly higher at T1 (*M* = 1.88; *SD* = 1.68) as compared to T2 (*M* = .96; *SD* = 1.49), *t*(23) = 2.298, *p* = .031. All other differences in PSI between T1 and T2 were not significant. Changes in other outcome variables between T1 and T2 were also not significant. For CGI severity of illness, at T1 and T2, a minimum of 1 and a maximum of 6 was observed (T1: *M* = 3.16 ± 1.59; T2: *M* = 3.00 ± 1.41; *p* = .868). The GDS-15 revealed a minimum of 1 and a maximum of 12 (T1) and 11 (T2) points (T1: *M* = 5.66 ± 3.48; T2: *M* = 5.50 ± 3.62; *p* = .809).

Current suicidal thoughts at T1 were reported by *n* = 11 patients (34.38%). Among patients with suicidal thoughts at T1, around half (*n* = 6) indicated that suicidality had developed or worsened over the last two weeks. At T2, *n* = 4 (16.7%) patients reported suicidal thoughts, while one patient had suicidal plans and another patient had a suicidal attempt. Patients with a history of suicidality were thoroughly questioned regarding current suicidal ideation. The need for further diagnostic was evaluated and – in case of necessity – the access to therapeutic interventions or acute treatment was ensured.

### Psychosocial Support

In the six months before the COVID-19 pandemic, all but one participant (*n* = 31) reported receiving some kind of psychosocial support. The most frequently used support was outpatient treatment in a psychiatric institution (81.3%). At T1, the percentage of people receiving psychosocial support decreased as compared to the six months before T1. This was significant for day clinics (*p* = .004), outpatient treatment (*p* = .012), physiotherapy (*p* = .008) and sports (*p* < .001). For most psychosocial supportive measures, percentage of usage increased (e.g. from 6.3% to 25% for sports) or remained the same from T1 to T2. Only the percentage of people in inpatient treatment fell (18.8% to 8.3%). See Table 4 for an overview of the psychosocial support system of the elderly patients before the pandemic, at T1 and at T2.

**Table 4:** Psychosocial support system of patients

	Six months before T1 (*n* = 32)	T1 (April/ May 2020) (*n* = 32)	T2 (August 2020) (*n* = 24)
Psychosocial support	*n* (%)	*n* (%)	*n* (%)
Any support	31 (96.9%)	29 (90.6%)	24 (100%)
Inpatient treatment	8 (25%)	6 (18.8%)	2 (8.3%)
Day clinic	9 (28.1%)	0*	1 (4.2%)
Outpatient treatment	26 (81.3%)	17 (53.1%)*	16 (66.7%)
Psychotherapy	2 (6.3%)	1 (3.1%)	2 (8.3%)
Rehabilitation	1 (3.1%)	0	0
Psychiatric nursing service	0	1 (3.1%)	1 (4.2%)
Nursing service	3 (9.4%)	2 (6.3%)	4 (16.7%)
Physiotherapy	17 (53.1%)	5 (15.6%)*	6 (25%)
Art/occupational therapy	12 (37.5%)	7 (21.9%)	8 (33.3%)
Group therapy	3 (9.4%)	2 (6.3%)	1 (4.2%)
Self-help-group	3 (9.4%)	1 (3.1%)	1 (4.2%)
Sports	19 (59.4%)	2 (6.3%)*	6 (25%)
Social counselling	5 (15.6%)	3 (9.7%)	3 12.5%)
Social support, e.g. case work	1 (3.1%)	1 (3.1%)	1 (4.2%)
Other	6 (18.8%)	2 (6.3%)	4 (16.7%)

*Significant change (*p* < .05) as compared to six months before T1

A percentage of 53.85% of patients who had been in outpatient treatment before the pandemic could continue their treatment because sessions were held on the phone. There were few other reports of technical solutions to provide continuing treatment, such as receiving materials for arts therapy via mail.

At T2, the number of patients receiving outpatient treatment via phone calls had decreased to *n* = 6, i.e. face-to-face sessions were more frequent (T1: *n* = 3; T2: *n* = 10). McNemar’s test revealed that these changes were non-significant.

Concerning the demand for psychosocial support at T1, more than half of patients (53.13%) reported a wish to do sports, followed by arts and occupational therapy (40.63%), physiotherapy (37.50%), treatment in a psychiatric outpatient facility (28.13%) or a day clinic (18.75%) and psychotherapy (15.63%). There were isolated reports (*n* < 4) of other demands. At T2, demand for an opportunity to do sports was still highest with 29.17%, followed by physiotherapy (25%), psychotherapy (20.83%), and arts or occupational therapy (16.67%). Other reports of demand at T2 were *n* < 4. The changes in demand were non-significant.

## Discussion

To our knowledge, this is the first longitudinal study in elderly psychiatric patients reporting a significant association between psychosocial burden and both depression and severity of illness as well as significant differences in PSI regarding social contact during the COVID-19 pandemic. The findings may provide information for policy makers in the current and potential future crises on how to best support this population.

### Psychosocial Impact

A negative PSI of the pandemic was reported by most participants which is in line with effects of the pandemic on the general population, as shown by a recent meta-analysis [38].

Patients with higher symptom burden of depression reported a more negative PSI of the pandemic. More specifically, at T1, patients with higher depression scores indicated a more severe PSI. Moreover, at T1, patients who were rated to be more severely ill were also more negatively impacted by the pandemic. Severity of illness showed a significant positive correlation with PSI due to financial issues during the pandemic.

One possible explanation is that depression in the elderly is linked to lower resilience [39], i.e. a decreased ability to protect one’s mental health when confronted with stressors [40]. Thus, patients with depression may be less able to handle isolation, uncertainty and other demands during the pandemic. A significant difference between psychiatric patients and healthy controls during the COVID-19 pandemic and higher levels of PTSD, anxiety, depression, stress and insomnia had been shown [41].

In addition, patients in a depressed state may be biased towards a more negative self-evaluation of their current situation than during a non-depressive condition.

We were able to verify the impact of the pandemic situation on patients with psychiatric conditions and further show a link between illness severity and PSI. This outlines the importance of offering effective psychosocial support to elderly patients with pre-existing psychiatric conditions. It should be noted however that results could also be explained by a negative bias in self-reports among patients suffering from depression.

### Social Contact

Significant inverse correlations were found between social contact behavior and negative PSI: people with more social contact felt less impacted by being member of a risk group, by difficulties with medical care access, by restrictions in leisure, social activities and consumption.

It has previously been shown that social contact can reduce psychosocial distress during a pandemic [42]. This is critical, given that containment measures such as social distancing reduce social contacts. These measures can cause distress and traumatization [12].

Surprisingly, patients with close social contact had a significant higher general PSI than patients with rare social contact at T1 and general PSI values in those with regular social contact were lowest. Elderly people may suffer from loneliness or non-integrated social networks [43]. Thus, patients being used to cultivating social contact and closeness to dependents may therefore be more severely affected by reduced contact and altered existing relationships/routines.

### Changes between T1 and T2

T2 was set in August 2020 when the Robert Koch Institute described the development of the 7-day incidence as “concerning” [44]. During this time, the number of new reported daily SARS-CoV-2 infections reached over 2000 cases for the first time since end of April. While no severe lockdown measures were in place at that point, certain pandemic-related restrictions were ongoing, such as the recommendation to wear a mask and socially distance. We did not find a significant reduction in general PSI nor in main mental health outcome variables between T1 and T2. Indeed, the high number of drop-outs at T2 (*n* = 12) has to be taken into consideration where several patients reported to be too unstable to continue participation.

To some extent, PSI may have decreased at T2 considering lower infection numbers compared to T1 as well as the patient’s adaption to the pandemic situation and containment measures. Decreasing PSI values due to the restriction themselves support this statement – supposedly due to habituation and an establishment of helping systems.

Alternatively, PSI may have increased due to partly ongoing restrictions and re-arising infection numbers in August 2020. Patients may have been impacted by expectation of reinstallation and intensification of policies, leading to a deterioration of PSI as compared to T1. This explanation seems plausible as many patients stated that they were afraid of a “second wave” and worried about new restrictions and “recklessness” of people who did not adhere to regulations.

The missing difference between the two timepoints may suggest antagonizing effects of both refuted hypotheses.

A previous study during the COVID-19 outbreak also revealed no significant change in PSI one month after the first survey. The mean scores for depression, anxiety and stress also did not change [45]. Even though data was collected in different phases of the COVID-19 pandemic, PSI appears to not remarkably change over a period of a few months. This may be caused by a fundamental feeling of uncertainty and even be strengthened considering the older age of participants in the presented study with more critical needs for consistency and lack thereof during the pandemic.

Follow-up studies after complete withdrawal of containment measures are needed to assess differences in PSI during and after the ongoing pandemic.

At T1, suicidal thoughts were more frequent than at T2, whereas suicidal plans and attempts were only reported at T2. Suicidal ideation in the context of containment measures has been described previously and is likely to be linked to the pandemic situation [46, 47].

### Psychosocial Support

Psychosocial support decreased due to the pandemic. This was reflected in the reported demand for supportive measures: a majority of patients wished to do sports again and many reported a demand for arts and occupational therapy, physiotherapy, treatment in an outpatient facility or day clinic and psychotherapy. While the psychosocial support system had grown at T2, there was still a high demand for sports, physiotherapy, psychotherapy and arts or occupational therapy. Being a study conducted in current and former patients of a psychiatric hospital, these results portray a group of people dependent on psychosocial support due to pre-existing psychiatric illnesses. However, the results also show that elderly patients have diverse interests and are open to receive help and support.

During the pandemic, programs for psychosocial crisis intervention have evolved, also using remote strategies such as telephone or internet [48]. Moreover, multidisciplinary teams have been built in order to improve mental health of people being psychosocially affected during the pandemic [49]. This was also reflected in our sample: many patients were able to continue outpatient treatment via phone calls. However, a positive effect of information and communication technologies on loneliness in elderly people could not yet be shown [50], revealing the complexity of improvement by modern communication tools in this group even if they are available.

### Limitations

Several limitations have to be considered in interpretation of our results.

While telephone interviews may enable patients to talk more freely, they also carry the risk of misunderstandings. Moreover, only retrospective questioning was conducted concerning the time before the pandemic, possibly reducing accuracy of reports.

Further, inclusion of patients may have been dependent on the ability to participate in interviews. Patients with comparatively better psychosocial well-being may be over-represented. In line with challenges of recruiting higher aged patients and the rapidly evolving COVID-19 crisis at T1, the presented study consists of a relatively small cohort. This may explain why apparent changes in psychosocial support were non-significant.

Overall, due to the small sample size and the high number of drop-outs at T2, statistical power is limited. Hence, the study has to be considered as a pilot study. Future studies with a larger number of patients in the context of patients and controls are needed to re-evaluate and confirm the presented results.

Generally, as the COVID-19 crisis is a rapidly evolving scientific issue, the discussed literature is biased towards studies being published rather at the beginning of the pandemic. Cited literature may originate primarily from countries where the pandemic started to spread, possibly reducing generalizability.

## Conclusion

In summary, our study in elderly patients with psychiatric disorders found patients with depression, higher psychiatric illness severity and less social contact to be more affected by the current COVID-19 pandemic. Utilization of psychosocial support decreased during the pandemic despite a high demand for support reported by elderly patients.

These results highlight the challenges for elderly psychiatric patients during the pandemic. In addition to proneness to somatic illness and severe sequelae of SARS-CoV-2 infection, these individuals are more likely affected by an impaired psychosocial situation.

Patients’ needs should be considered in political decisions and clinical management to ensure well-being of this patient group throughout this ongoing as well as future pandemics.

## Data Availability

M. Seethaler and S. Just had full access to all of the data in the study and take responsibility for the accuracy of the data analysis and the integrity of the data.

## Abbreviations

COVID-19: Coronavirus Disease-2019
SARS-CoV-2: Severe Acute Respiratory Syndrome Coronavirus 2
ICU: Intensive Care Unit
MERS-CoV: Middle East Respiratory Syndrome-related Coronavirus
PTSD: Posttraumatic Stress Disorder
PSI: Psychosocial Impact
DSM-5: Diagnostic and Statistical Manual of Mental Disorders
GDS-15: German Geriatric Depression Scale
OCD: Obsessive-Compulsive Disorder
CGI: Clinical Global Impression – Severity Scale
